# Reduced emissions from deforestation and forest degradation (REDD): a climate change mitigation strategy on a critical track

**DOI:** 10.1186/1750-0680-4-10

**Published:** 2009-11-13

**Authors:** Michael Köhl, Thomas Baldauf, Daniel Plugge, Joachim Krug

**Affiliations:** 1 Institute for World Forestry, University of Hamburg, Leuschnerstr. 91, 21031 Hamburg, Germany; 2 Institute for World Forestry, von Thünen-Institute, Leuschnerstr. 91, 21031 Hamburg, Germany

## Abstract

**Background:**

Following recent discussions, there is hope that a mechanism for reduction of emissions from deforestation and forest degradation (REDD) will be agreed by the Parties of the UNFCCC at their 15th meeting in Copenhagen in 2009 as an eligible action to prevent climate changes and global warming in post-2012 commitment periods. Countries introducing a REDD-regime in order to generate benefits need to implement sound monitoring and reporting systems and specify the associated uncertainties. The principle of conservativeness addresses the problem of estimation errors and requests the reporting of reliable minimum estimates (RME). Here the potential to generate benefits from applying a REDD-regime is proposed with reference to sampling and non-sampling errors that influence the reliability of estimated activity data and emission factors.

**Results:**

A framework for calculating carbon benefits by including assessment errors is developed. Theoretical, sample based considerations as well as a simulation study for five selected countries with low to high deforestation and degradation rates show that even small assessment errors (5% and less) may outweigh successful efforts to reduce deforestation and degradation.

**Conclusion:**

The generation of benefits from REDD is possible only in situations where assessment errors are carefully controlled.

## Background

According to estimates by the International Panel on Climate Change (IPCC) 1.6 billion tons of carbon are released annually by land-use change activities, of which a major part results from deforestation and forest degradation [1]. The Stern Report [2] pointed out that nearly one-fifth of today's total annual carbon emissions come from land-use change, most of which can be traced back to tropical deforestation. Deforestation is generally understood as the direct human-induced conversion of forest land to non-forest land [3], while forest degradation is according to Intergovernmental Panel on Climate Change (IPCC) [3] the direct-human induced long-term loss of forest carbon stocks in areas which remain forest land. Among the causes of degradation are the collection of fuelwood, selective logging, forest fires, grazing or shifting cultivation [4].

For the 2008-2012 commitment period of the Kyoto Protocol (KP) avoiding deforestation was discussed as a CDM activity and rejected. Leakage was seen as uncontrollable at the project level. In 2005 at the Eleventh Session of the Conference of Parties (COP 11) to the United Framework Convention on Climate Change (UNFCCC) Papua New Guinea together with 8 other developing countries proposed a new agenda item "reducing emissions from deforestation in developing countries" at a national level. This was the start of the process for considering reducing emissions from deforestation and forest degradation in developing countries (REDD) as a mitigation option for those countries. Following the related discussions and proceedings, there is hope that a REDD mechanism will be agreed by the Parties of the UNFCCC at their 15th meeting in Copenhagen in 2009 as an eligible action to prevent climate changes and global warming in post-2012 commitment periods.

A country participating in a future REDD mechanism of the UNFCCC has to demonstrate substantial capacities for monitoring and accounting emissions from forest carbon stocks. Thus a reliable framework for measuring, reporting and verification is vitally needed to ensure the integrity and credibility of REDD efforts in general and REDD in the post-2012-agreements to be approved in Copenhagen in particular. While approaches for monitoring, reporting and verification, as well as potential financing mechanisms for a provision of appropriate incentives have been discussed intensively [4,5], little attention has been paid so far to the fact that uncertainties associated with the estimation of forest area and carbon stock changes have a fundamental impact on accountable carbon credits and the cost-benefit ratio.

In this study we present error sources associated with the monitoring of above ground forest biomass and carbon stock in the scope of REDD and discuss the implications of uncertainties on the reliable minimum estimate (RME) that is requested for IPCC reporting.

## Results

Applying the conceptual framework described in the Methods, we demonstrated that monitoring costs required for a sound determination of RMEs may outweigh a substantial proportion of potential financial benefits that could be generated for emission certificates under national REDD-schemes.

A country that intends to benefit from the adoption of a REDD-regime, needs to proof that deforestation and forest degradation in a current commitment period is smaller than it was in the periods before. Accountable carbon credits, Ĉ_t2REDD_, are obtained by subtracting the real carbon stock at time 2, C_t2real_, from the carbon stock expected under the baseline scenario, C_t2BL_, which is derived from past deforestation and degradation rates. The larger the difference the more carbon credits are generated.

To illustrate the effect of the inclusion of uncertainties in REDD estimates, we selected five countries that hold small to large forest areas and show low (-0.23%) to high (-10.57%) deforestation rates (Table 1[6,7]).

**Table 1:** Characteristics of the countries selected for the case study (taken from FAO's Global Forest Resources Assessment [6])

Country	Category*	Forest area 2005 [1000 ha]	Carbon stock [tC/ha]	Forest area development, based on 2000-2005) [1000 ha/year]	Carbon stock 2005 [Mt]^+^	Δ_BL_ [%]	Carbon stock 2010, according to baseline [Mt]
Bolivia	HFMD	58,740	66.63	-270	3,914	-2.30	3,824

Cameroon	MFMD	21,245	63.05	-220	1,340	-5.18	1,270

Gabon	HFLD	21,775	137.11	-10	2,986	-0.23	2,979

Indonesia	HFHD	88,495	50.10	-1,871	4,434	-10.57	3,965

Madagascar	LFLD	12,838	186.09	-37	2,389	-1.44	2,355

HF = high forest area, LF = low forest area, MF = medium forest area

MD = medium deforestation rate, LD = low deforestation rate, HD = high deforestation rate

* according to Griscom [7]

^+ ^it is assumed that carbon stock 2005 (C_t1_) is the RME

For each country the rate of deforestation between 2000 and 2005 was utilised to predict the carbon stock at the end of a five year period between 2005 and 2010, C_t2BL _under a business-as-usual (BAU) development. Reductions of the business-as-usual deforestation and degradation by 10, 30, 50 and 75 percent were simulated for each country - stipulating that a country was able to reduce its business-as-usual deforestation and degradation by 10%, 30%, 50% or 75% - and the respective carbon stocks at time 2, C_t2real_, calculated. The corresponding differences between C_t2real _and C_t2BL _reflect different levels of accountable carbon credits generated by REDD, Ĉ_t2REDD_.

The estimation of carbon stocks is subject to several error sources, including sampling errors, assessment errors, or prediction errors from models. Those error sources can be random or systematic in nature and are combined to the so-called total error of the estimate. The total error defines an interval around the estimate which quantifies the uncertainties associated with the estimate. For reasons of conservativeness the lower bound of the interval is defined as the reliable minimum estimate (RME). For calculating the accountable carbon credits generated by a REDD regime the RME of C_t2real _is used in order to reflect uncertainties. Including errors generally reduces the amount of accountable emission reduction due to avoided deforestation and degradation.

**Figure 1:**
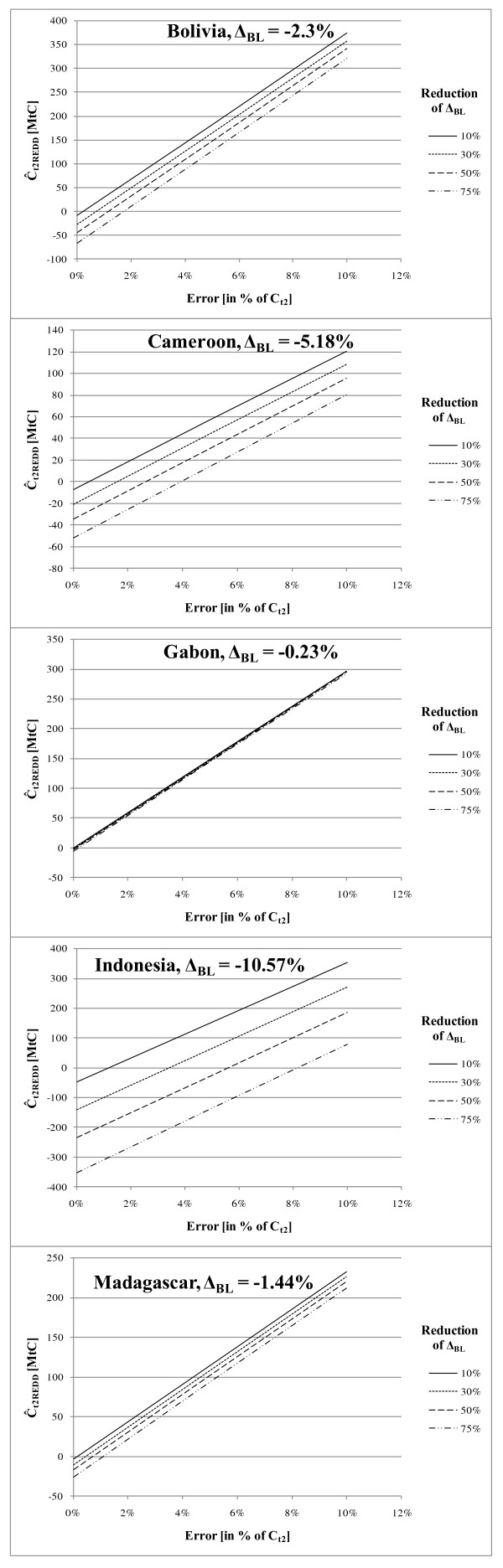
Graphs of resulting Ĉ_t2REDD _(in tC*10^6^) for five selected countries in relation to error at time 2, (E_t2_) for different reduction scenarios for Δ_BL _showing the effect of total error and deforestation and degradation rates on carbon credits; positive numbers display emissions, negative numbers removals.

To show the effect of errors, the reliable minimum estimates (RME) of C_t2real _under the four reduction rates were calculated for 0 to 10% total error and used as reference for calculating the respective carbon credits, Ĉ_t2REDD_. Figure 1 presents the results of the simulation study. The accountable carbon credits are plotted for the four reduction scenarios over different levels of total error (0 to 10%). Positive numbers for Ĉ_t2REDD _display CO_2 _emissions to the atmosphere, negative numbers display CO_2 _reductions.

Taking a 5% total error for the estimation of the carbon stock at time 2, C_t2real_, only Indonesia (deforestation rate = -10.57%) would qualify for generating carbon credits, if a reduction of the deforestation and degradation-rate of at least 50% were reached. According to studies from Fuller et al. [8], Gertner and Köhl [9] or Waggoner [10] total errors larger than 5% are most likely to occur.

Among the selected countries Gabon shows the lowest deforestation and degradation rate (-0.23%). The reduction of the deforestation and degradation rate will lead to only minor gains in total carbon stock at time 2 due to the low deforestation rate. Even negligible total errors of carbon estimates at time 2 render the generation of accountable carbon credits impossible. The commitment to a REDD regime would put a country like Gabon (high forest area, low deforestation rate) in a situation, where - despite the efforts in further reducing the already low deforestation and degradation rate - carbon emissions from forests would need to be reported.

Even for a reduction of the deforestation and degradation rate by 75% Madagascar (low forest cover, deforestation rate = -1,44%) would be excluded from REDD benefits when total errors are larger than 1% and Bolivia (high forest area, deforestation rate = -2,3%) would need to provide estimates with a total error less than 2% in order to generate benefits from REDD.

Cameroon, a country with medium forest area and medium deforestation rates (-5,18%), would need to assess the carbon stock at time 2 with an error smaller than 4% when the deforestation and degradation rate is reduced by 75%.

The adoption of a REDD regime should be most suitable for countries with high forest areas and high deforestation rates. However, Figure 1 shows for the example of Indonesia (high forest area, deforestation rate = -10,57%) that even a 50% reduction of the deforestation and degradation rate would render a total error of roughly 6% necessary in order to generate benefits from REDD.

The results of the simulation study suggest that even small errors result in situations where no carbon credits can be generated (Figure 1). The effect of the total error on the RME is much larger than the effect of different reduction rates of deforestation and degradation. Total errors larger than 5%, which are realistic in extensive forest carbon surveys [8-10], exclude most national REDD-regimes from generating benefits. The simulation study indicates that countries with medium or low deforestation and degradation-rates are not in a position to generate benefit from REDD when the uncertainties of carbon stock estimates are included in calculations as requested in a REDD certification process.

## Discussion

The generation of carbon credits by introducing a REDD scheme becomes critical when the principle of conservativeness and assessment errors are considered in the monitoring and reporting process. As shown by theoretical considerations and a simulation study the total error associated with carbon estimates can outweigh efforts to reduce deforestation and degradation. Introducing a REDD-regime in situations where the error structures of the assessment and monitoring system are unknown, may result in critical situations; only a minor amount of carbon credits could be generated or - even worse - emissions from forestry need to be reported, even when the country committed itself to REDD and was successful in reducing carbon losses from deforestation and degradation.

When REDD is considered as an economic approach to conserve forest ecosystems in developing countries, the benefits generated from the implementation of a REDD system need to be larger than the benefits from deforestation. A prerequisite is the estimation of activity data and emission factors with high certainty. Due to the superior role of the total errors associated with carbon stock estimates, significant efforts need to be taken to reduce uncertainties by sound monitoring and reporting systems. However, those systems are expensive and may in many countries not or only partially be covered by the generated benefits (see [11] for inventory cost estimates). Monitoring costs under a sound construction of RMEs compensate a substantial proportion of the financial benefits that could be generated for emission certificates under national REDD-schemes.

## Conclusion

In further studies on approaches to capture deforestation and degradation special focus needs to be taken to the quantification of the total survey error. Feasibility studies without sound non-sampling and sampling error assessments are useless for decisions about the "optimal" REDD inventory concept. We recommend that especially countries in the readiness phase, which have not yet developed appropriate capacities, carefully study the effects of the principle of conservativeness in preparing for REDD. For those countries capacity building for implementing sound carbon monitoring systems is urgently needed in order to turn efforts in reducing deforestation and forest degradation into benefits generated by REDD. However, countries with already low deforestation rates will most likely not benefit from REDD.

## Methods

### Assessment of emissions from deforestation and degradation

In forests there are five major carbon pools [3]: (1) above ground biomass, (2) below-ground biomass, (3) dead wood, (4) litter, and (5) soil organic matter. The avoidance of deforestation and forest degradation aims at the maintenance of carbon in the living biomass, for which reason the most practical monitoring approach is to concentrate on the assessment of the carbon pool "above ground biomass".

Monitoring and reporting of deforestation and degradation requires the assessment of two components [3]:

- changes in forest area over time, and

- changes in the average carbon stock per unit area over time.

The quantification of changes requires assessments at successive occasions or the availability of models that allow for the extrapolation of data from one point in time to another. The total loss of forest carbon stock in a given period and area is the sum of two components: (1) the product of average carbon stock per unit area times the forest area changed from forest land to other land use in the respective period, and (2) the reduction of average carbon stock in areas that remain forest land. In order to increase the reliability of estimates, the area of forests can be subdivided in several classes indicating different levels of carbon stock decrease or degradation.

Area changes can be either assessed by field-based sample surveys or by remote sensing techniques. The latter are generally more cost efficient and provide not only point estimates (i.e. forest area) but spatially explicit data in mapped format. Remote sensing data are often utilised to separate the total forest area into different sub-groups or strata, such as occurring forest types, e.g. broadleaf, tropical moist and tropical dry. In addition probabilistic approaches can be used to complement the forest classification by risk factors that describe the probability of degradation, based on proxies such as past level of human interventions, accessibility or population density. Remote sensing techniques enable the detection of deforestation, especially on large areas. More difficult is the quantification of forest degradation, where even substantial removals of biomass do not necessarily lead to a pronounced reduction of canopy cover. Only far advanced stages of forest degradation can be detected by remote sensing techniques.

Carbon stock changes can be quantified by various methods. A straightforward approach is to utilise default values from secondary sources such as from IPCC [3]. Estimates based on default values can be subject to great uncertainties, as they may not reflect the true country specific values. A more reliable alternative is to apply country specific data on degradation to individual forest types or risk categories. The most reliable estimates of carbon stock changes are obtained by sample based field assessments on successive occasions. On in-situ sample plots individual trees are measured and biomass and carbon stock are calculated on the plot level. Upscaling procedures expand plot data to area related estimates [12]. Those assessments provide sound and sensitive estimates of changes in forest biomass and degradation activities.

Recommendations on methods and default values for assessing carbon stocks and emissions are provided by the IPCC Good Practice Guidance [3] and Greenhouse Gas Inventory Guidelines [13]. For calculating changes in average carbon stock per unit area the IPCC [3,13] proposes two approaches:

(1) the stock difference method that makes reference to traditional forest resource assessments and calculates changes in average carbon stock per unit area as the difference between carbon stock at time 2 and time 1, and

(2) the gain-loss method that builds on the understanding of carbon uptake by forests (tree growth) and carbon release by anthropogenic activities such as timber removals, fuelwood gathering, sub-canopy fires or grazing.

Forests may be stratified into sub-areas with different degradation intensities in order to increase the reliability of the estimated carbon losses.

As there are substantial differences between countries regarding the capacities and implemented assessment systems for monitoring, reporting and validating carbon stock changes, the IPCC-guidelines provide three tiers of detail for reporting.

- Tier 1 offers the simplest to use alternative that utilises globally-available activity data (e.g. on deforestation rates). Equations and default values (e.g. emission and stock change factors) are provided by IPCC [13]. Tier 1 reporting is recommended for countries with limited availability of country-specific data. However, Tier 1 estimates do not qualify for reporting in the scope of REDD due to the large error rates, which are in the range of ± 50% [11].

- Tier 2 utilises country- or region-specific data for the most important land-use categories. Emission factors and activity data show a higher temporal and spatial resolution than those used for Tier 1.

- Tier 3 uses high order methods including models and inventory measurement systems that are tailored for the country specific circumstances. The methods are driven by high resolution activity data and may include comprehensive field sampling repeated at regular time intervals as well as GIS-based systems to analyse land-use data.

Moving to higher Tiers reduces the uncertainty of estimates but increases the complexity and cost of the utilised monitoring and reporting systems. In order to be flexible for implementation on the country level the good practice guidance (GPG) [3,13] allows for a combination of Tiers, e.g. Tier 2 for changes in average carbon stock and Tier 3 for land use changes.

### Uncertainties

The implementation of REDD as a mitigation option in the context of UNFCCC needs to ensure the credibility of estimated emissions and removals from deforestation and degradation. In its 28^th ^session the Subsidiary Body for Scientific and Technological Advice (SBSTA) was concerned with methodological issues concerning the implementation of REDD and stated that "means to deal with uncertainties in estimates aiming to ensure that reductions in emissions or increases in removals are not overestimated" need to be further considered [14]. Uncertainties evolve from the assessment and estimation methodologies applied. In REDD those are mainly linked to the assessment of deforestation and degradation areas (activity data, AD) and the carbon stock changes in those areas (emission factor, EF).

The estimation of AD and EF is subject to two major error types: sampling errors and non-sampling errors [15]. Sampling errors arise from inferring from a subset (i.e. the sample) of the population to the whole population. The size of sampling errors can be controlled by the survey design and the size of the sample. Non-sampling errors encompass all other sources of errors involved in a survey, which can be the faulty application of definitions, classification errors, measurement errors, errors arising from the application of functions and models, calculation errors, or frame errors (i.e. the sample population is different from the target population). Different types of errors can be quantified by giving their precision, accuracy, or bias.

- Precision refers to the size of deviations in the estimate of a population parameter in repeat application of a sampling procedure. The standard error or confidence interval quantifies precision. Increasing the number of observations increases the precision of a statistical estimate.

- Accuracy refers to the size of deviations between an observed value and the true value. Thus, if the true value of a population parameter is known then the accuracy of a survey estimate can be defined as the deviation between the estimate and the true value.

- Bias is directly related to the accuracy of an estimate and refers to systematic errors that affect any sample with the same constant error.

**Figure 2:**
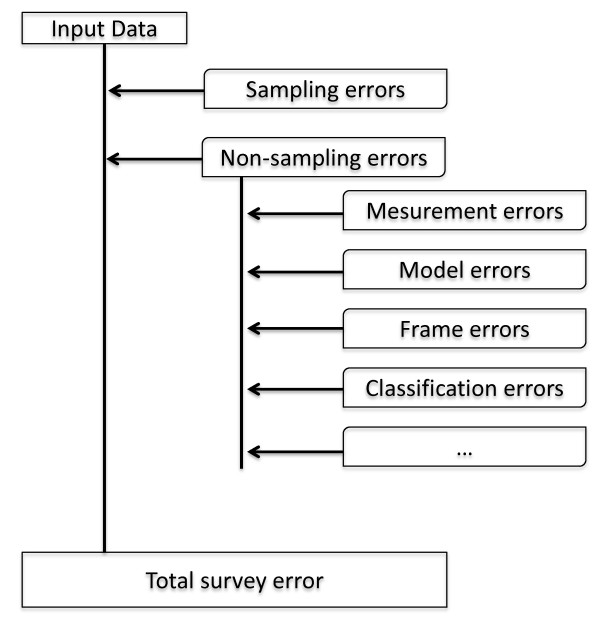
**Total survey error and error budget**.

The IPCC Good Practice Guidance [3,13] suggests the 95%-confidence interval to quantify the uncertainty of estimates. In this context the use of the term confidence interval is not very specific, as from a puristic statistical point of view the confidence interval is related to sampling errors only. The total survey error quantifies all error sources associated with an estimate. This can be realised via an error budget (Figure 2).

The Mean Square Error (MSE) is a useful measure of the total error, as it combines sampling errors with the square of the bias. For unbiased estimators MSE and precision are asymptotically identical.

The quantification of AD requires estimates of forest area changes over time. Where remote sensing techniques are used, the uncertainty embedded in estimating changes between two points in time is influenced by the map accuracies at both occasions and the magnitude of changes. Fuller et al. [8] discuss the measurement of land-cover change over time and present a statistical approach to quantify the reliability of change estimates. They show that for 10-class maps the accuracy at both times needs to be 99% to detect a smaller than 20% change with a 90% reliability. Thus it is rather idealistic to expect the sensitive detection of area changes by multi-temporal analysis of remote sensing data.

**Figure 3:**
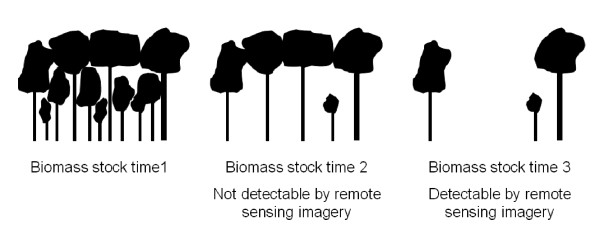
**Different status of forest degradation and potential of detection by optical remote sensing techniques**.

The detectability of degradation by remote sensing data is another critical issue. Especially in natural forests stands in the tropics and subtropics, which are characterised by heterogenic vertical stand structures and contiguous canopy covers, degradation can only be detected, when the formerly closed canopy cover is dissolved (Figure 3).

EFs are to be quantified by in-situ assessments in forest stands, which follow the rules of probabilistic sampling theory. Carbon stock of trees is quantified via above ground volume or biomass figures. As those cannot be assessed directly on standing trees they are estimated via volume or biomass functions, which utilise tree measurements such as diameters, tree heights or crown parameters as independent variables. Where volume estimates are available they can be converted into biomass estimates by biomass expansion factors (BEF). Biomass estimates are transferred into carbon stock estimates by applying biomass-carbon conversion factors, which depend on the wood density of the respective tree species and tree components.

The EF-estimates are subject to a series of error sources, including measurement errors and function errors. A serious problem is introduced by frame errors. Assessments of a limited set of field plots may not be representative for the entire tree species, forest types, ecosystem regions and disturbance levels within a country [16,17]. IPCC [3] presented figures for above ground biomass, which show a large range of variability. For example, in wet tropical forests the possible range of values covers 34% to 248% of the average. This shows that currently a high level of uncertainty is associated with the quantification of above ground biomass stock.

### The principle of conservativeness

Grassi et al. [18] propose to use the principle of conservativeness in order to "address the potential incompleteness and high uncertainties of REDD estimates". The principle of conservativeness has already been reflected in several UNFCCC documents, for example in the context of afforestation and reforestation activities under the Clean Development Mechanism (CDM) [19,20].

According to Grassi et al. [18] the completeness principle depends on "the processes, pools and gases that need to be reported and on the forest-related definitions". Both, uncertainties and incompleteness need to be considered for quantifying carbon stock changes under REDD activities. In the context of the assessment of changes in soil carbon, the IPCC-Good Practice Guidance suggests using the Reliable Minimum Estimate (RME) to address uncertainties. The RME was introduced by Dawkins [21] as the minimum quantity to be expected with a given probability and served as a surrogate for the lower bound of a confidence interval.

**Figure 4:**
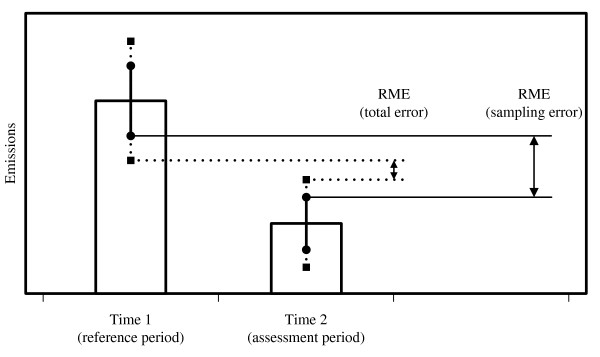
**Reliable Minimum Estimate (RME) in terms of confidence interval (sampling error, bold lines) and total survey error (dashed lines)**.

The principle of the RME can be expanded from a mere sampling error perspective to the concept of total survey errors and transferred to the assessment of forest carbon stock changes. The RME is the difference between the lower bound of the error interval at the reference period (time 1) and the upper bound of the error interval at the assessment period (time 2) and can be treated as a conservative estimate that qualifies for accounting. Where the confidence interval is used, only sampling errors are considered and the resulting magnitude of emission reduction is considerably larger than for an RME that is taking into account on the total survey error (Figure 4).

**Figure 5:**
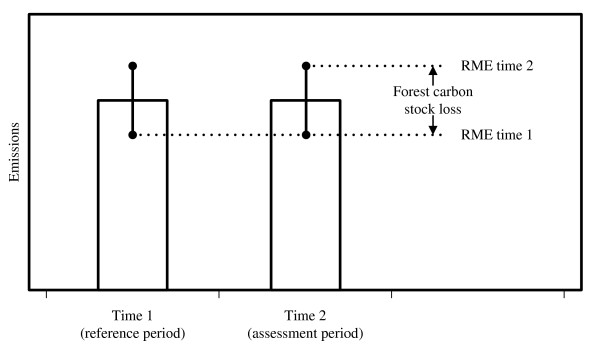
**Effect of conservativeness principle for a country maintaining its forest carbon stock, RME = Reliable Minimum Estimate**.

The principle of conservativeness is a wise recommendation for countries that are still in the readiness phase, but have not yet implemented a sound REDD inventory concept. However, the principle of conservativeness might result in a counterproductive situation where the forest area within a country is maintained or only slightly decreased. As the RME at time 1 would be (considerably) lower and the RME at time 2 higher than the estimated (unchanged) forest area, a forest area loss would need to be reported (Figure 5). Or, in other words, a country without deforestation activities would only be able to report an unchanged forest area under the principle of conservativeness, when the area of afforestation activities has the same size as the difference between the RMEs at time 1 and time 2. However, under such conditions it would not be wise for a country to introduce a REDD-regime.

### Benefits from REDD

A country is able to generate benefits under a REDD regime, when the deforestation and degradation in a reporting period is reduced compared to the respective baseline. Such a baseline can be established in different ways; here the baseline is assumed to be a business-as-usual scenario, which is obtained by a linear extrapolation of past deforestation and degradation rates. The amount of benefits generated depends not only on the committed reduction of deforestation and degradation, but on the reliability of the estimated change as well.

**Figure 6:**
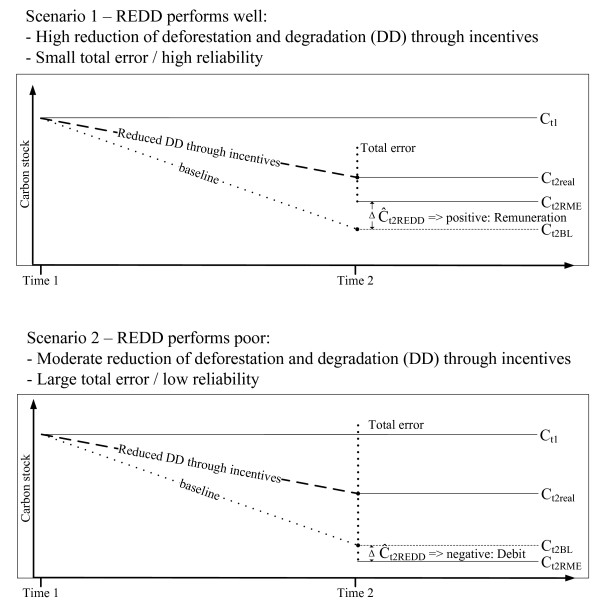
**Relationship of reduction of deforestation and forest degradation (DD), total error and reliable minimum estimate (RME), and their contribution to the values of carbon stock at time 1 (C_t1_), virtual carbon stock at time 2 according to a baseline scenario (C_t2BL_), real carbon stock at time 2 (C_t2real_), carbon stock at time 2 qualifying for accounting (C_t2RME_) and difference of C_t2BL _and C_t2RME _(Ĉ_t2REDD_)**.

Figure 6 illustrates the relation between reduced deforestation and degradation and reliability of its estimates. In scenario 1 a country with high reduction of deforestation and degradation is shown. The RME-principle is applied to address the uncertainty of estimates in the figures to be reported. As the reduction rate is considerably larger than the associated uncertainty in estimates, the resulting amount of emission reduction qualifies for the generation of credits.

In scenario 2 the situation is different. Here the associated errors in estimating the reduced emissions by avoided deforestation and degradation are larger than the emission reduction itself. Therefore a country under scenario 2 would not qualify for benefits from emission reduction, as it failed to provide evidence that the committed reduction of deforestation and degradation was met.

The potential benefit generated by a REDD regime at time 2 is subject to the amount of carbon stock qualifying for accounting, C_t2REDD_, and the prices per ton of CO_2_. C_t2REDD _is calculated as the difference between the virtual carbon stock according to a baseline scenario, C_t2BL_, and the real carbon stock at time 2, C_t2real_.(1)

Positive numbers for C_t2REDD _represent emissions to the atmosphere (the source function); negative numbers represent removals from the atmosphere (the sink function of forests).

C_t2BL _can be expressed in terms of the carbon stock at time 1, C_t1_, and its change indicated by the baseline, Δ_BL_.(2)

where

C_t2BL _= expected carbon stock at time 2 according to the baseline

C_t1 _= carbon stock at time 1; it is assumed that C_t1 _is the RME

Δ_BL _= proportional change between time 1 and time 2 according to the baseline, Δ_BL _= {-1.1}, where negative values indicate a decrease of the C-stock, e.g. by deforestation or degradation, and positive values an increase, e.g. by afforestation or forest growth.

The carbon stock observed at time 2, C_t2real_, is given by(3)

where

Δ_real _= proportional real change between time 1 and time 2, Δ_real _= {-1.1} where negative values indicate a decrease of the C-stock, e.g. by deforestation or degradation, and positive values an increase, e.g. by afforestation or forest growth.

With eq. (2) and (3) the carbon stock qualifying for accounting given by eq. (1) can be rephrased:(4)

In eq. (1) to (4) no error components are included. The amount of carbon qualifying for accounting needs to include estimates of the underlying uncertainties. Thus C_t2RME_, which is constrained by the RME at time 2, has to be reported:(5)

where

E_t2 _= error of the estimated carbon stock at time 2, C_t2real_

Replacing C_t2real _by C_t2RME _in Eq. (1) yields a REDD estimate Ĉ_t2REDD_, which incorporates uncertainties for the estimated carbon stocks at time 1 and time 2,(6)

Eq. (6) illustrates the drivers of the amount of carbon that generates benefits. E_t2 _is controlled inter alia by the inventory concept applied, Δ_real _reflects the efforts undertaken to reduce deforestation and degradation, and Δ_BL _points to the carbon stock that would be achieved under business-as-usual interventions.

**Figure 7:**
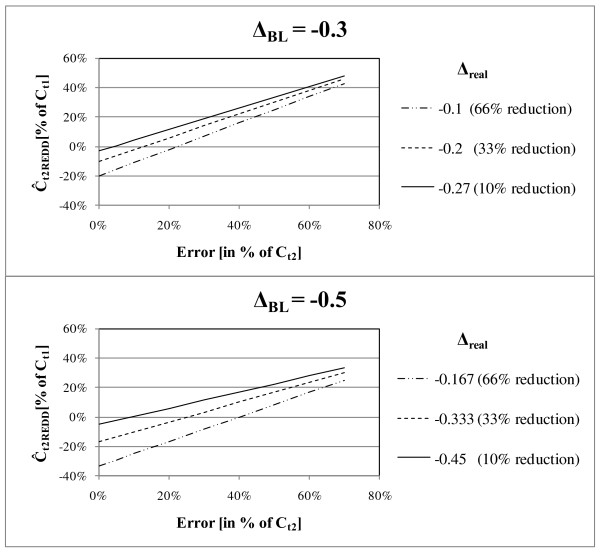
**Graph of resulting Ĉ_t2REDD _(in percent of carbon stock at time 1, C_t1_) in relation to error at time 2, (E_t2_) for different real deforestation rates (Δ_real_); different baseline changes (Δ_BL_): upper graph: Δ_BL _= -0,3; lower graph, Δ_BL _= -0,5; positive numbers display emissions, negative numbers removals**.

The effect of errors on the RME and the impacts for benefits generated by REDD are illustrated in Figure 7. For errors, E_t2_, ranging from 0 to 70 percent of C_t2real_, Ĉ_t2REDD _was calculated in percent of the carbon stock at time 1, C_t1_. Changes between time 1 and time 2 according to the baseline were chosen to be 30 and 50 percent (Δ_BL _= {-0.3; -0.5}). The real changes, Δ_real_, are between 10 and 45 percent, resulting in an emission reduction of 10 to 66 percent.

It is obvious that benefits can only be generated where real changes, Δ_real_, are smaller than the changes according to the baseline, Δ_BL_, as Ĉ_t2REDD _becomes negative. However, the amount of benefits generated depends on the error associated with the carbon stock estimates, E_t2_. The functional relationship between Δ_BL_, Δ_real _and E_t2 _indicates that the smaller the difference between Δ_BL_, and Δ_real_, the smaller E_t2 _has to be in order to generate benefits. For example, an error E_t2 _smaller than 12 percent of the real carbon stock observed at time 2 is required to achieve accountable carbon credits for a baseline change, Δ_BL_, of 30 percent and a real change, Δ_real_, of 20 percent (Figure 7). As large errors corrupt the generation of accountable carbon credits by REDD, reasonable care has to be exercised in implementing a sound assessment and reporting system.

## Competing interests

The authors declare that they have no competing interests.

## Authors' contributions

MK conceived of the study, participated in its design and coordination, and drafted the manuscript. TB and DP carried out the study and performed the statistical analysis. JK participated in the design of the study. All authors read and approved the final manuscript.
